# Scaling up mental health care and psychosocial support in low-resource settings: a roadmap to impact

**DOI:** 10.1017/S2045796020001018

**Published:** 2020-11-26

**Authors:** Mark J. D. Jordans, Brandon A. Kohrt

**Affiliations:** 1 Research & Development, War Child Holland, Amsterdam, the Netherlands; 2 Amsterdam Institute of Social Science Research, University of Amsterdam, Amsterdam, the Netherlands; 3 Division of Global Mental Health, Department of Psychiatry & Behavioral Sciences, The George Washington University

**Keywords:** Community mental health, evidence-based psychiatry, health service research, minority issues and cross-cultural psychiatry, quality of care

## Abstract

**Aims:**

Despite recent global attention to mental health and psychosocial support services and a growing body of evidence-support interventions, few mental health services have been established at a regional or national scale in low- and middle-income countries (LMIC). There are myriad challenges and barriers ranging from testing interventions that do not target priority needs of populations or policymakers to interventions that cannot achieve adequate coverage to decrease the treatment gap in LMIC.

**Method:**

We propose a ‘roadmap to impact’ process that guides planning for interventions to move from the research space to the implementation space.

**Results:**

We establish four criteria and nine associated indicators that can be evaluated in low-resource settings to foster the greatest likelihood of successfully scaling mental health and psychosocial interventions. The criteria are relevance (indicators: population need, cultural and contextual fit), effectiveness (change in mental health outcome, change in hypothesised mechanism of action), quality (adherence, competence, attendance) and feasibility (coverage, cost). In the research space, relevance and effectiveness need to be established before moving into the implementation space. In the implementation space, ongoing monitoring of quality and feasibility is required to achieve and maintain a positive public health impact. Ultimately, a database or repository needs to be developed with these criteria and indicators to help researchers establish and monitor minimum benchmarks for the indicators, and for policymakers and practitioners to be able to select what interventions will be most likely to succeed in their settings.

**Conclusion:**

A practicable roadmap with a sequence of measurable indicators is an important step to delivering interventions at scale and reducing the mental health treatment gap around the world.

## Introduction

Mental health care in low- and middle-income countries (LMIC) is receiving increasing attention in research, practice and policy. Recent years have seen several responses to the large unmet mental health needs due to unavailable human and financial resources in LMIC. The Sustainable Development Goals (SDG) include a clear reference to mental health and the World Health Organization (WHO) is enacting a Comprehensive Mental Health Action Program. With the 2018 first-ever inter-ministerial summit on global mental health in London and a second in 2019 in the Netherlands focusing on mental health in humanitarian settings, the need for mental health care in LMIC has made its way to policy makers. Researchers have evaluated models of mental health services delivered by non-professionals, and synthesised current evidence in a series of recent reviews (van Ginneken *et al*., 2013; Singla *et al*., 2017; Kohrt *et al*., 2018; Purgato *et al*., 2018). However, with all these positive developments, few mental health interventions and programmes have been brought to scale.

A major challenge is knowing what works where, for whom and how. The questions inform what interventions should be scaled and how to assure that they are effective at scale. There is variability in outcomes among and within LMIC settings even when the same intervention is used (Fuhr *et al*., 2019; Sikander *et al*., 2019; Dorsey *et al*., 2020). Yet, it is not feasible to perform extensive adaptations and run new randomised controlled trials (RCT) in every single setting where a new mental health or psychosocial intervention is going to be deployed. That said, the variability in outcomes means that one cannot assume that interventions will be effective – and what may be going wrong when they are not effective – when scaled beyond the original effectiveness RCT (Kohrt *et al*., 2020). Unfortunately, there is not a minimum set of guidelines for what to monitor in the scale-up process to measure if and why or why not an intervention is working.

In a time that mental health services are increasingly being incorporated in policy and planning (Patel *et al*., 2018), we think a roadmap is needed to provide guidance for governments and international agencies that are implementing such services both for new and existing services. Such a roadmap would outline a practicable trajectory for testing and implementing mental health and psychosocial support interventions, using a minimum set of criteria that maximise scalability and impact combined with tools to operationalise and benchmark the criteria. To meet this need, we propose a ‘roadmap to impact’ for scaling up mental health care and psychosocial support in low-resource settings.

## Roadmap to impact

**Fig. 1.**
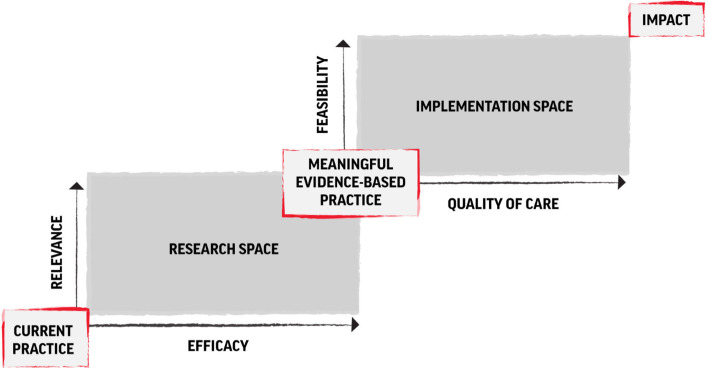
Roadmap to impact – research and implementation trajectory to achieve scale. *Note*: This figure has been adapted from the version published in Jordans *et al.* (2018).

The ‘roadmap to impact' model bridges research and practice, two sides of a coin that have traditionally been rather divided. Unfortunately, the interventions most implemented in practice tend to be those with the least research evidence, and the findings from research have had limited impact on practice (Tol *et al*., 2011). Therefore, the roadmap connects the research space with the intervention space, to create a pathway from evidence to practice with measurable indicators along the trajectory (see Fig. 1). First, the research space involves intervention-level research that establishes relevance and effectiveness and expands the evidence-base for task-shifted care. Second, the implementation space involves system-level work that transfers meaningful evidence-based interventions to large-scale impact (Jordans *et al*., 2018). In the implementation space, quality and feasibility criteria need monitoring, beginning with benchmarks established in the research phase that are continuously refined through real-world delivery. We will explain the roadmap by describing the four criteria that need to be met to successfully scale, and we provide guidance, by way of example frameworks, on how each criterion can be evaluated.

### The research space

The starting point for any intervention is demonstrating its relevance (criterion #1) within a given LMIC setting. Given the growing diversity of potential mental health and psychosocial support interventions that vary by treatment duration, training and supervision requirements, and target group or condition, it is important to avoid a one-size-fits-all approach to services. Relevance needs to precede effectiveness, because an effective intervention is unlikely to receive buy-in for scaling if there is not a recognisable benefit at the community and policy levels. Relevance can be demonstrated by two indicators: population need and fit with the culture and context (see Table 1). (a) Population need is the degree to which the intervention addresses actual and current mental health needs within the target population. The *Assessing Mental Health and Psychosocial Needs and Resources: Toolkit for Humanitarian Settings* is an example framework that provides resources for determining what would be relevant in a particular context, but the advised approaches have not been systematically used in low-resource settings outside of humanitarian context (WHO and UNHCR 2012). There needs to be a clear demonstration of need, as well as mapping of current services to be sure that the proposed intervention does not duplicate existing resources. Likelihood for scaling will be greatest when a gap is being filled. (b) The second indicator is the ‘fit’ or compatibility with culture and context. Ensuring a match between context and intervention has demonstrated to result in superior treatment outcome (Chowdhary *et al*., 2014), though this association is not undisputed (Cuijpers *et al*., 2018). An example framework for adapting psychological interventions to the culture and context addresses cultural concepts of distress, treatment components and treatment delivery (Heim and Kohrt, 2019). An argument has been made that being culturally appropriate is not enough, but rather that successful interventions need to be ‘culturally compelling’ in that the intervention taps into key motivations or drivers for individual, family and community roles within a given culture or context (Panter-Brick *et al*., 2006), i.e. that interventions address ‘what matters most’ for both beneficiaries and providers (Kohrt *et al*., 2020*b*).

**Table 1.** Domains and indicators for roadmap to impact

Criteria	Indicator	Research space	Implementation space
*Relevance*	Population need	Document population need when selecting type of intervention	Document population needs in new areas when scaling
	Cultural and contextual fit	Identify modifications needed to content, delivery agents, community engagement, etc.	Establishment of, and engagement with, service users, community advisory boards, other stakeholders
*Effectiveness*	Mental health outcome	Comparative outcomes between standard of practice and novel intervention	Routine monitoring of outcomes
	Mechanism of action	Identification of active ingredients associated with positive outcomes	Selective monitoring through self-report tools, passive data collection on mobile devices
*Quality*	Adherence	Establish fidelity levels at which the intervention is effective	Structured observation of in-service sessions and periodic monitoring through ongoing supervision
	Competence	Establish minimum competency level for effectiveness	Structured observation using role plays and periodic monitoring through ongoing supervision or in-session observations
	Attendance	Establish minimum attendance needed for effectiveness	Recording attendance in programmatic monitoring
*Feasibility*	Coverage	Document recruitment and retention rates for eligible participants	Documentation in national health surveys; health systems records
	Cost	Incremental cost-effectiveness ratios establishing added value of novel intervention	Documentation of ongoing delivery costs

*Note.* The darker shaded cells represent the primary application of the indicator within the roadmap, whereas the lighter shaded cells represent the secondary application of the indicators within the roadmap.

Only once the criterion of relevance is met, can one move towards the criterion of effectiveness (criterion #2). The study of the effectiveness of mental health services has been a pillar for psychological treatment research for several decades. Effectiveness studies have exponentially increased in LMIC resulting in a large body of trials in LMIC (Patel *et al*., 2018). (a) The first indicator of effectiveness is a positive effect size for the mental health outcome of interest. In an umbrella review of 129 primary studies from ten meta-analyses, representing 22 623 participants, Barbui *et al*. (2020) demonstrate that there is robust evidence for psychosocial interventions for adults with depression, schizophrenia, as well as PTSD in humanitarian settings. For children, the evidence base is much smaller with suggestive evidence only for children with disruptive behaviour or with PTSD in humanitarian settings (Barbui *et al*., 2020). This means that evidence is accruing, but also that there is still some distance to go for a package of interventions with a solid evidence base, covering multiple mental health conditions and a wider age range. Consequently, there is a need for more trials demonstrating effectiveness. For policy makers, cost-effectiveness needs to assessed for interventions for which the current evidence-base is still weak, especially mental health promotion and prevention programmes, and child and adolescent treatments. An example framework to establishing the evidence is a five-step research process used for the evaluation of WHO's newly developed interventions, which involves formative research, a feasibility trial combined with a qualitative process evaluation, and a definitive trial combined with a qualitative evaluation (Bryant *et al*., 2017). (b) The second indicator of effectiveness is a measured mechanism of action. Besides RCTs demonstrating effectiveness, there is also a need for greater attention to evaluating hypothesised mechanisms of action and to conduct dismantling studies (i.e. measuring separate pieces of interventions) to better understand the active ingredients determining how and why interventions are effective. Mechanisms of action can be measured with self-report scales, but it is preferable to use behavioural observation or capitalise on behavioural data that can, for example, be acquired through passive sensing of mobile digital devices, such as smartphones. With a better understanding of underlying mechanisms of change, one can adjust delivery of the interventions to maximise the benefits of interventions.

### The implementation space

For those interventions that do meet both of the aforementioned criteria (relevance and effectiveness), the next challenge is *how* such interventions can be implemented at scale. We now enter the implementation space in the model. This domain of study, implementation science, is gaining momentum in LMIC (Means *et al*., 2020), and pertains to the study of how evidence-based interventions can be implemented to have the intended results among a large proportion of people in need of care. Attention to the relevance of an intervention can be accomplished through ongoing engagement with service users and community advisory boards, and routine monitoring of client outcomes can shed light on if and for whom an intervention is working. However, the emphasis in the implementation space is on criteria along two axes: (1) quality of care, and (2) feasibility. These two criteria are crucial to monitor the implementation of an evidence-based intervention at scale and understand why an intervention may not be working in a specific setting or with a specific population.

Therefore, in order to assure that the results demonstrated in a well-controlled trial are also achieved in everyday practice, we propose systematic assessment of quality of care (criterion #3), which we have operationalised using three minimum indicators – (a) adherence, (b) competence and (c) attendance. These are defined as the extent to which a service provider has the knowledge and skill required to deliver a treatment to the standard needed for it to achieve its expected effects (competence) and the extent to which a psychological treatment was delivered well enough for it to achieve its expected effects (adherence) (Fairburn and Cooper, 2011). In addition, participants need to receive enough of the intended content (attendance). With this minimum set of indicators, we argue, a programme can assess quality of care at scale; and if adequate levels of competence, adherence and attendance have been obtained, the positive client-level outcome, as demonstrated in the research space, can be assumed, rather than needing to always be measured, which is typically not feasible at scale. For example, a service provider can be highly competent, however, if implementing without adequate adherence to an intervention protocol, then we cannot assume a positive outcome. Similarly, if a provider is meticulously following the intervention protocol, but does not have the core therapeutic skills and competencies, then again positive outcomes cannot be assumed. The same goes for service providers who demonstrate high levels of competence and adherence but who are working in a context where participants are unable to consistently attend and participate in care. If, however, adherence, competence and attendance are all adequate, we assume that we can rely on previously established research findings.

This thinking is commonly advocated by proponents of empirically supported treatments, suggesting that the key to transporting effectiveness findings in everyday clinical settings is ensuring high levels of therapist competence and adherence (Collyer *et al*., 2020). However, for this approach to work in LMIC when working with non-specialists, there is a need for each of these indicators to be validated or benchmarked, such that we know what level of adherence, competence and attendance needs to be obtained to substantiate an assumption of effectiveness. Validation of competence indicators is currently underway in an effort to guide the scaling of psychological treatments by the WHO and partners (Kohrt *et al*., 2020*a*). This is based on prior work done to develop new tools that allow for the assessment of competencies that are common across all mental health interventions. These new tools, ENhancing Assessment of Common Therapeutic Factors (ENACT) for adults (Kohrt *et al*., 2015*a*), and Working with children – Assessment of Competencies Tool (WeACT) for children (Jordans *et al*., under review) have been developed specifically for feasible use with non-specialists in low-resource settings. In the original tool development studies in Nepal and Gaza, we demonstrated that such competency assessment, using standardised role-plays and life observations, captures changes before and after training, and can be used by multiple raters with sufficient reliability (Kohrt *et al*., 2015*b*; Jordans *et al*., under review). The tools have also since been used to evaluate the competencies of mental health service providers in several different low-resource settings (Kohrt *et al*., 2018; Rahman *et al*., 2019).

Benchmarks for adherence, competence and attendance need to be established initially in the research space. Either through reporting of details related to adherence, competence and attendance in RCTs, or through separate validation studies. However, it is within the implementation space that these benchmarks should be refined because there will be considerably greater variation in these three indicators in real-world settings. For example, the rigorous procedure in RCTs is unlikely to lead to the inclusion of providers with low competency. While in routine practice, there will likely be a range of competency levels among providers in public and private health and service institutions. Similarly, in most trials, procedures are in place to promote attendance, and the same level of effort is beyond the scope of most national health systems. Therefore, adjusted benchmarks will likely emerge through real-world implementation of the intervention. These benchmarks can be especially useful for guiding incremental changes in government implementation strategies to improve existing services.

The monitoring of the three indicators and comparison against minimum benchmarks also allows for quality improvement. Tracking levels of adherence, competence and attendance within any given area of programme provides supervisors with an overview of which indicators fall below the validated thresholds. For example, if a group of service providers consistently scores low on certain competencies or specific treatment components, or in case of a trend in drop-outs, then supervisors can remediate this with more targeted and tailor-made solutions. In turn, we hypothesise that such targeted quality monitoring is more cost-effective than approaches that do not have such a data-driven approach. An example framework that brings together tools and knowledge for the assessment of quality of care is WHO's Ensuring Quality in Psychological Support (EQUIP) program (https://www.who.int/mental_health/emergencies/equip/en/) (Kohrt *et al*., 2020*a*).

This brings us to the last axis of the model, feasibility (criterion #4), and the associated indicators – (a) coverage and (b) cost. Because even if interventions are relevant and effective, and quality is maintained, population-level impact is only achieved if a large enough proportion of those for whom the intervention is intended are actually reached. The level of uptake is expressed as contact coverage, and defined by Tanahashi (1988) as the ratio between the number of people who have contacted the service and the size of the target population. Reaching a certain level of coverage needs to be determined for scale-up to be deemed successful. An example framework is the Goldberg-Huxley model, which describes a process of help seeking for people with mental disorder along a set of filters that need to be addressed to maximise coverage (Goldberg and Huxley, 1980). An application of that can be found in the Program for Improving Mental Health Care (PRIME), which evaluated the integration of mental health into primary health care (Lund *et al*., 2012; Jordans *et al*., 2019*b*). The programme demonstrated that population-level change in contact coverage was not achieved in some of the settings– even after significant efforts to make services available (Nakku *et al*., 2019; Shidhaye *et al*., 2019). Besides such supply-side approach, demand-side drivers will therefore need to be addressed in order to actually achieve significant changes in contact coverage. Community-level awareness raising, stigma-reduction approaches and proactive case detection may be strategies that increase demand (Eaton *et al*., 2018; Jordans *et al*., 2020). Contact coverage can be calculated with the attendance data (see criterion 3c) combined with epidemiological data on the prevalence of the condition the intervention is targeting. Finally, for true scalability of impact, the cost of implementation of the intervention needs to be acceptable for settings with limited resources for mental health services. This means that besides evaluating the cost-effectiveness for interventions, the feasible implementation of interventions at scale will need to include monitoring of costs-per-person against set targets specific for certain settings and population (Chisholm *et al*., 2017; Chisholm *et al*., 2020). Although trials can establish incremental cost-effectiveness ratios, the actual implementation costs can vary significantly from what was estimated under trial conditions.

In brief, we argue for monitoring the adherence, competence and attendance of relevant evidence-based interventions against validated thresholds or standards, combined with targets for contact coverage and per-person cost. This forms the minimum set of criteria to guide the process of scaling mental health intervention and achieve population-level impact.

## Discussion

To date, myriad implementation frameworks have been developed as descriptive, prescriptive, explanatory, or predictive heuristics for the traditional translation pipeline from efficacy to dissemination and implementation, e.g. Replicating Effective Programs (REP), Consolidated Framework for Implementation Research (CFIR), and Reach, Efficacy, Adoption, Implementation, and Maintenance (RE-AIM). Our proposed model provides added value to these frameworks by identifying a minimum set of specific criteria that planners of mental health services can use to plot where a programme is in along the pathway to scalability. It also provides guidance about how to achieve greater impact, giving example frameworks and tools that can be used in practice for achieving each of the criteria. Moreover, the model can be used to address trade-offs between optimizing impact while taking into account constraints on issues related to relevance, effectiveness, quality and feasibility. Existing implementation science frameworks, especially prescriptive frameworks, can be used to elucidate how to move from one region of the roadmap to impact to another region. Finally, the presented model emphasises *practicability* by proposing a set of concrete and measurable criteria and indicators that have been tried and tested in LMIC, and at scale it can do so with data that can be entirely obtained from the service delivery agents – therefore not relying on individual-level data from participants to demonstrate impact.

One strength of the model is that the quality of care indicators reduces data collection to a minimum. At the same time, we acknowledge that such data collection requires commitment and resources of mental health care planners. In an effort to maintain quality of services, this is likely a worthwhile investment. Moreover, previous efforts to use routine data collection for mental health services have demonstrated feasibility in several low-resource settings (Jordans *et al*., 2019*a*). More broadly, successful application of this model is dependent on commitment from governments or international agencies. This entails a policy context that prioritises evidence-based mental health care, and the allocation of resources to implement the services at scale, as well as the monitoring framework consisting of the criteria put forward in this paper. National and global investments will be required to develop the infrastructure for these indicators and technical expertise to manage data collection and interpretation of information. Otherwise, adding indicators without a system of analysis and action would risk detracting energy from already stretched-thin public mental health systems. Consequently, the application of the model should be part of a larger effort of mental health system strengthening.

We propose that for such an approach to be operational, a central repository is established where data are stored and accessible for policymakers and practitioners across the global mental health field. In fact, we envision that the validated quality criteria (benchmarks) can become endorsed inter-agency standards. Any agency scaling up evidence-based treatment would therefore strive to achieve these standards, but also agree to report against them in the data repository. If such data, at aggregate level, are made open access it allows for monitoring of overall scaling efforts across geographic areas, across interventions, across organisations. This could draw upon approaches and lessons learned from similar systems for registering RCTs in a public repository (e.g. ClinicalTrials.gov, ISRCTN), data repositories for specific funders (e.g. NIMH Data Archive), collaborative databases for specific conditions (e.g. Autism Brain Imaging Data Exchange) and evidence-based interventions that are searchable by implementation characteristics (e.g. Research-Tested Intervention Programs (RTIPs) for cancer, rtips.cancer.gov).

There are several limitations to the proposed model. First, while the notion of evidence-based care is increasingly being adopted, one can argue that we are still too far removed from having a solid evidence-base in LMICs. Cuijpers *et al*. (2018), synthesizing the literature on treatments for depression, argue that the effects that have been demonstrated in the literature tend to be over-estimated – provokingly asking whether psychological treatments work at all (Cuijpers *et al*., 2019). More attention to strengthening the evidence base is clearly needed (the research space) – for example, for children and adolescents, as also shown in the review by Barbui *et al*. (2020). We believe that the research agenda can simultaneously emphasise the study of how evidence-based intervention can be implemented at scale (the implementation place), rather than consecutively. The model provides a framework for guiding that research. Second, one might ask whether the level of standardisation of interventions and quality indicators across participants and across cultural settings is possible. As much as interventions will need to be adjusted to new cultural contexts, without changing any of the key working mechanisms, we propose that the quality indicators equally will need to be adjusted for the different settings, including renewed validation studies. Third, the model does not provide an exhaustive overview of the process and indicators involved in scaling, and as such does not do justice to all the complexities involved in scaling. For example, we have not included indicators assessing the political commitment, or to assess quality of care we acknowledge that attendance alone is an insufficient indicator of dosage, as that will also depend on participants' level of engagement with the intervention. We have aimed to keep a minimum set of indicators that is measurable at scale.

## Conclusion

This paper provides a framework to guide the implementation of evidence-based mental health and psychosocial interventions at scale in real-world settings, using a streamlined set of criteria to maximise impact at population level. If adequate quality of implementation of evidence-based treatment is obtained (through the assessment of competence, adherence and attendance criteria relying on validated cut-points), combined with adequate feasibility (through the assessment of cost and coverage criteria using a priori set targets), then this provides a foundation for positive outcomes at scale. Scaling without these minimum standards is unlikely to translate evidence-based research into public health impact to reduce the mental health treatment gap around the world.

## Data Availability

No data were used for this article.
